# Electrochemical Oxidation of Organic Pollutants in Aqueous Solution Using a Ti_4_O_7_ Particle Anode

**DOI:** 10.3390/membranes13050521

**Published:** 2023-05-17

**Authors:** Andrey Kislyi, Ilya Moroz, Vera Guliaeva, Yuri Prokhorov, Anastasiia Klevtsova, Semyon Mareev

**Affiliations:** Membrane Institute, Kuban State University, 149 Stavropolskaya St., 350040 Krasnodar, Russia; andrey.kislyi@mail.ru (A.K.); klevtsovaanastasiia1995@gmail.com (A.K.)

**Keywords:** electrochemical advanced oxidation processes, anodic oxidation, particle anode, electrochemical flow cell, organic contaminants, hydroquinone, benzoic acid

## Abstract

Anodes based on substoichiometric titanium oxide (Ti_4_O_7_) are among the most effective for the anodic oxidation of organic pollutants in aqueous solutions. Such electrodes can be made in the form of semipermeable porous structures called reactive electrochemical membranes (REMs). Recent work has shown that REMs with large pore sizes (0.5–2 mm) are highly efficient (comparable or superior to boron-doped diamond (BDD) anodes) and can be used to oxidize a wide range of contaminants. In this work, for the first time, a Ti_4_O_7_ particle anode (with a granule size of 1–3 mm and forming pores of 0.2–1 mm) was used for the oxidation of benzoic, maleic and oxalic acids and hydroquinone in aqueous solutions with an initial COD of 600 mg/L. The results demonstrated that a high instantaneous current efficiency (ICE) of about 40% and a high removal degree of more than 99% can be achieved. The Ti_4_O_7_ anode showed good stability after 108 operating hours at 36 mA/cm^2^.

## 1. Introduction

Electrochemical advanced oxidation processes (EAOPs) are recognized as next-generation technologies for water treatment due to their high efficiency, simplicity of design and high degree of mineralization of organic pollutants [1,2,3,4,5]. Anodic oxidation (AO) is the most common among EAOPs. This method allows the oxidation of organic compounds in aqueous solutions without the addition of chemical reagents [6]. The AO process proceeds along two main pathways: direct electron transfer (DET) and with the participation of intermediate electron carriers or mediated electron transfer. Anode materials for AO are usually divided into two groups: “active” and “non-active” [7,8]. In the case of “active” anodes, the formed hydroxyl radicals immediately interact with the anode material. Then, the resulting higher oxide is further involved in the oxidation of organic compounds. The materials of “non-active” anodes do not interact with the formed radicals. Thus, the formed radicals remain physically adsorbed on the anode surface [9]. The surface of “non-active” anodes with high oxygen evolution potential (OEP) [10] facilitates the formation of the reactive species of hydroxyl radicals (HO^•^), capable of oxidizing most complex organic pollutants [11,12,13] at current densities higher than the limiting value, which leads to a decrease in fouling. Briefly, the oxidation of organic compounds by HO^•^ radicals can be written as follows:

(1) $$M+H_{2}O\to M(HO^{•})+H^{+}+\bar{e}$$

(2) $$\begin{array}{l} R+M(HO^{•})\to \mathrm{degradation}\;\mathrm{by}-\mathrm{products}\to \\ \to nCO_{2}+mH_{2}O+\mathrm{other}\;\mathrm{oxidation}\;\mathrm{products} \end{array}$$

One of the main directions in AO development is the design of new promising “non-active” anode materials that increase the oxidation process efficiency and its stability and reduce energy consumption.

Substoichiometric titanium oxide (Ti_4_O_7_) is a recognized effective electrode material [12,14,15] due to its relatively high electrical conductivity, chemical stability, high OEP, and relatively low production cost [16,17,18,19,20,21]. Both plate and porous anodes can be made from it.

Generally, plate electrodes are used for the AO process. A thick hydrodynamic diffusion boundary layer is formed near the surface of these electrodes, which limits the oxidation rate of organic compounds. The use of porous anodes makes it possible to reduce transport limitations [10] as they have improved hydrodynamics and large interphase, which makes them more accessible for the organics.

Ti_n_O_2n-1_ porous electrodes were first developed by Ebonex^®^ and used for the AO of trichloroethylene in 1999 [22]. Due to a small pore size (~1 μm) and the possibility of application in the flow-through mode, these and similar electrodes are called reactive electrochemical membranes (REMs) [23,24]. Over the next two decades, REMs were proven to provide high efficiency in the AO process [10]. Theoretical and experimental data show that in the case of compounds with low molecular weight, high current efficiency can be achieved [25,26]. However, the small pore size is also a weakness of REMs, as large polymerized organic particles and gas bubbles [27] get stuck in the pores and block them and shield the active surface of the anode. Thus, for example, in the case of phenol [25], polymerization leads to intrapore fouling, which significantly reduces the efficiency of the AO process.

It is possible to increase the size and control the shape of the pores of Ti_4_O_7_ anodes through 3D printing. In [28], the authors obtained electrodes with a pore size of approximately 1 mm. These electrodes allow the effectiveness of REMs to be maintained and reduce the probability of the formation of indelible insoluble deposits on the surface of the pores. Despite the fact that 3D printing makes the material morphology more controllable, including its shape, thickness and porous structure [29], its application on an industrial scale has not yet been realized.

Pressed particle electrodes may be considered as a type of porous electrode. They are made up of many small conductive particles that are interconnected. Particle electrodes can be a convenient alternative to electrodes obtained using 3D printing. Their granules can be of any shape and are easy to manufacture. Particle electrodes have been used for wastewater treatment for a long time [30,31]. The main advantages of such electrodes are high conductivity and a developed surface, which makes it possible to increase the efficiency of pollutant removal [31]. Most commonly, these electrodes are used in combination with plate electrodes, while the particle electrode granules fill the space between the cathode and the anode, creating a multitude of “microreactor electrolyzers” containing their own microanode and microcathode [32]. To the best of our knowledge, Ti_4_O_7_ is still not used as a material for particle anodes.

In this work, we studied the AO process using a particle anode made of Ti_4_O_7_. The study was carried out on model solutions of common organic pollutants at various flow rates and current densities.

## 2. Materials and Methods

### 2.1. Experimental Solutions 

**Figure 1:**
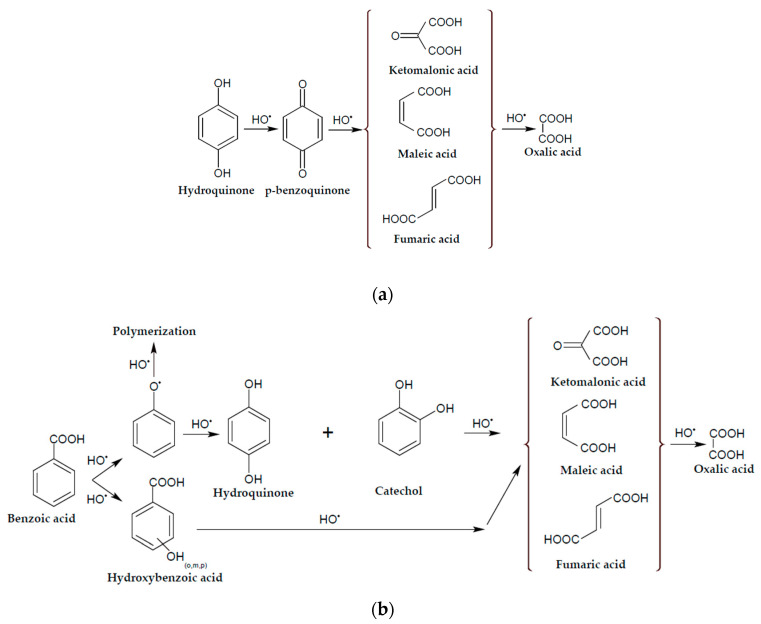
Possible degradation pathways for hydroquinone (**a**) and benzoic acid (**b**) [33,34].

The AO process of aqueous solutions containing the following organic compounds produced by “AO Vekton”, St. Petersburg, Russia, was studied: benzoic (98%), maleic (98%) and oxalic (98%) acids and hydroquinone (98%). The supporting electrolyte was 0.1 M sodium sulfate (anhydrous, 99%, “JSC LenReaktiv”, St. Petersburg, Russia). These substances were previously used in the AO process as a simple imitation of real effluents. In all experiments, distilled water (conductivity 1–5 μS cm) was used and obtained using a reverse osmosis laboratory water purification unit (“BMT Ltd.”, Vladimir, Russia). Possible degradation pathways for benzoic acid and hydroquinone are shown in Figure 1 [33,34]. The simple compounds (maleic acid and oxalic acid) are intermediates in these schemes, so their degradation pathways can also be followed:

### 2.2. Experimental Setup 

**Figure 2:**
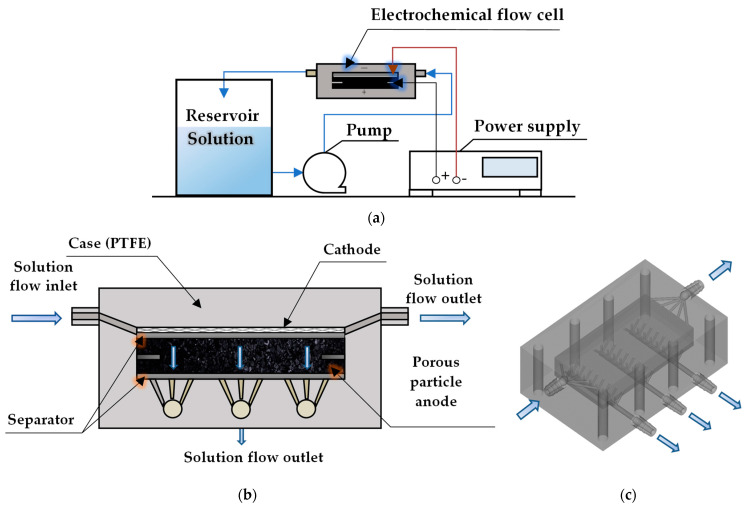
Schematic representation of the experimental setup (**a**) and EC-FC in 2D (**b**) and 3D (**c**).

The experimental setup consists of three main parts (Figure 2a): hydraulic system, hardware (electrical) and electrochemical flow cell (EC-FC). The hydraulic system provides solution storage (0.5 L reservoir), continuous supply of the model solution with constant flow rate inside the EC-FC using a membrane pump (0.1–1.1 L/min in this study) and sampling to analyze the carbon oxygen demand (COD) of the solution after AO. The hardware is used to maintain a direct current (DC) (or voltage) in the cell using an APS-5235 current supply (AKTAKOM, Moscow, Russia) and to measure the potential drop between the cathode and anode using an ABM-4552 voltmeter (AKTAKOM, Moscow, Russia). 

The EC-FC (Figure 2b,c) is a plate electrolysis chamber with a grid cathode and a porous particle anode divided by a mesh separator made of an inert material (polypropylene). The sealed case of the cell is made of polytetrafluoroethylene (PTFE), resistant to oxidation by most oxidants. It consists of three parts (10 mm thick), clamped between two stainless steel end plates by tie-rods. Silicone gaskets (1 mm in thick) are placed between the PTFE parts. The Ti_4_O_7_ particle anode (~110 g weight) is filled up into the lower part of the cell chamber (Figure 2b). The thickness of the anode layer is approximately 10 mm. The length and width of the cell chamber are 101 mm and 54 mm, respectively. The granule size of the Ti_4_O_7_ particle anode is 1–3 mm. The granules were manufactured by Shanghai Hy-Sailing Chemical Tech. Co. LTD, Shanghai, China. As mentioned above, the Ti_4_O_7_ particle anode is highly reactive with HO^•^ formation and DET and has high OEP. The cathode is platinized titanium (coating thickness 5.0 μm), which is inert with respect to the solution components and has a high electrical conductivity, made in the form of a rhomboid grid with a grid pitch of 7 mm. A polypropylene separator of 3 mm thick is placed between the anode and cathode. The thickness of the separator is chosen in such a way that it exerts a pressure on the anode to hold all the particles together and to ensure a good contact between them. The anode and cathode are connected to the power supply by means of platinum plates, which are located at opposite ends of the EC-FC.

The cell has one inlet and four outlets (the main one and three additional ones). The main outlet is used to take away the cathode and anode byproducts from the cell. The additional ones are used to provide organic compounds access to the entire pore surface of the anode. The flow rate of each additional outlet is eight times less than that of the main one. The solution from all the outlets is collected in one reservoir, well-mixed and pumped again into the EC-FC.

### 2.3. Experimental Procedure

The processing of an aqueous solution containing an organic compound was carried out as follows: Before every experiment, the EC-FC was washed with distilled water for 30 min. Then, the distillate was drained from the system, and a working solution containing organic compounds and a supporting electrolyte of a given concentration was pumped into the system. The total solution volume in the experimental setup was 500 mL. The COD solution was 600 mg/L (which is 159 mg/L for hydroquinone, 140 mg/L for benzoic acid, 363 mg/L for maleic acid, 1688 mg/L for oxalic acid). In all cases, the concentration of the background electrolyte, Na_2_SO_4_, was 0.1 M.

After 15 min, a constant current density in continuous mode was set in the system. Then, first sample of the solution (9 mL) for the analysis was taken from the reservoir, and after that, the procedure was as follows: first two hours—sampling every 30 min, after 2 h—every hour. The concentrations were analyzed by measuring the COD values using the photometric method. After 6 h, the electrical current was turned off.

### 2.4. Measurement Procedure 

The COD measurements using photometric method were carried out in accordance with the standard described in ISO 15705:2002 Water quality—Determination of the chemical oxygen demand index (ST-COD)—Small-scale sealed-tube method, using the following reagents: silver sulfate, Ag_2_SO_4_ (99%, “JSC LenReaktiv”, St. Petersburg, Russia) in concentrated sulfuric acid, H_2_SO_4_ (99%, “AO Vekton”, Russia) and potassium dichromate, K_2_Cr_2_O_7_ (99% “JSC Ekros”, St. Petersburg, Russia). The optical density of the samples was measured at a wavelength of 600 nm (UV-1800 spectrophotometer, TM ECOVIEW, St. Petersburg, Russia).

The analysis of the anode composition was carried out using X-ray fluorescence spectrometry for elemental analysis (Energy Dispersive X-ray Fluorescence Spectrometer EDX-8000, Shimadzu, Kyoto, Japan) and X-ray powder diffraction for phase identification (X-ray diffractometer XRD-7000, Shimadzu, Japan).

The optical images of anode granules before and after experiment were obtained using an SOPTOP CX40M microscope (Yuyao, Zhejiang, China). A field emission scanning electron microscope JSM-7500F (JEOL Ltd., Akishima, Japan) with INCA x-sight 6650 EDS microanalysis system (OXFORD instruments, Abingdon, UK) examined the morphology of the surface and the elemental analysis of the anode granules’ surfaces (before and after experiment).

The FT-IR spectra of anode granules before and after experiment were obtained using a Vertex-70 spectrometer attached to FT-IR-microscope HYPERION 2000 (Bruker Optics, Ettlingen, Germany).

## 3. Results and Discussion 

### 3.1. Elemental and Phase Analyses of Ti_4_O_7_ Granules

The X-ray powder diffraction analysis shows that the dominant phase in the material is substoichiometric titanium oxide, Ti_4_O_7_ (>98%) (see Supplementary Materials, Figure S4). The main impurities registered in the anode composition are shown in Table 1.

**Figure 3:**
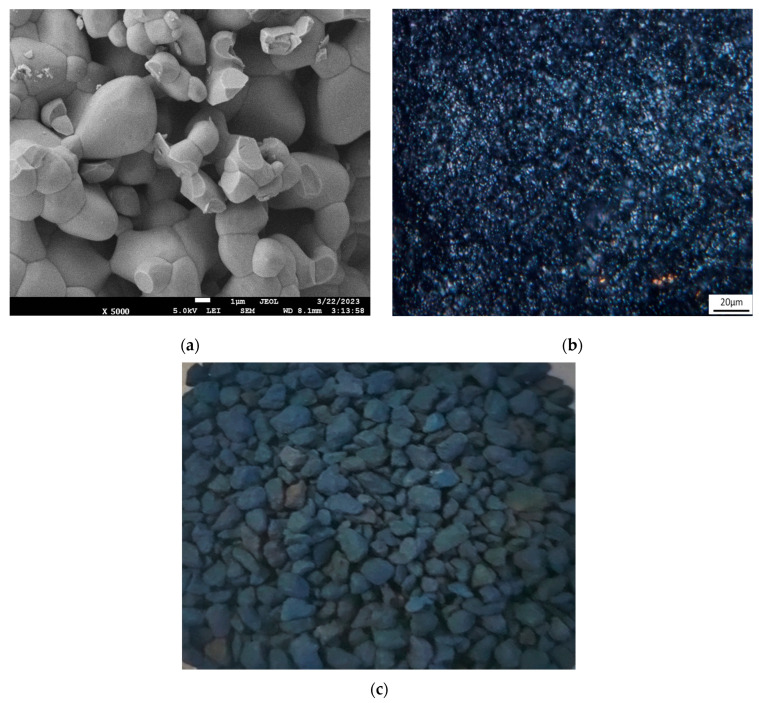
SEM (**a**) and optical images (**b**) of the anode granule surface and image of dry Ti_4_O_7_ particle anode (**c**).

The anode has a rough surface (Figure 3a), and its volume contains pores approximately 1 µm in size. Such a structure can ensure the sorption of organics and their subsequent mineralization both on the developed surface of the anode and in the volume of individual particles. 

### 3.2. COD of Studied Organics

The COD values in the samples taken at the outlet of the EC-FC immediately before the current is applied for all substances, except oxalic acid, are noticeably lower (5–10%) than the COD of the freshly prepared solution of 600 mg/L. This may be related to the experiment procedure. The anode and separators, after washing with distillate, which is always carried out between experiments, retain a small volume of water inside them (approximately 25 mL, 5%).

**Figure 4:**
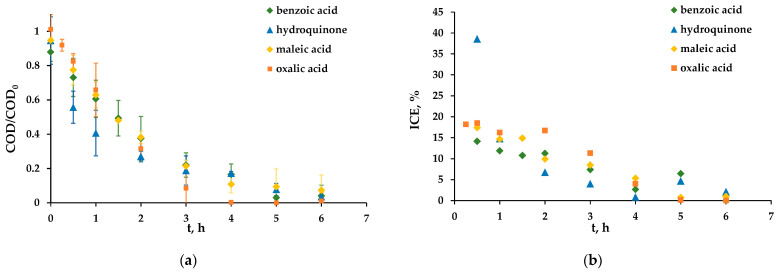
Dependence of the COD/COD_0_ of organics (**a**) and the ICE (**b**) on the experiment time at the current density of 36 mA/cm^2^.

The degradation kinetics of all studied organics (hydroquinone and benzoic, maleic and oxalic acids) at a current density of 36 mA/cm^2^ are shown in Figure 4a. According to the experimental data, we conclude that all the studied organics are oxidized at approximately the same rate. The exponential dependence of COD on its initial value on time, *t*, is clearly defined. It means that the system is under mass transfer control, and the current density, *i*, is higher than the limiting value, $i_{\lim}^{0}$, even at *t* = 0. Panizza et al. [35] proposed an equation, which may be used to calculate the time dependence of COD:

(3) $$COD(t)=\alpha COD_{0}\exp\left(-\frac{Ak_{m}}{V_{R}}t+\frac{1-\alpha }{\alpha }\right)$$

Where *V*_R_ is the solution (reservoir) volume (m^3^), *k*_m_ is the mass transfer coefficient in the electrochemical cell (m s^−1^), *A* is the electrode area (m^2^), COD_0_ is the initial value of COD (mol O_2_ m^−3^), *α* = $\frac{j_{appl}}{j_{\lim}^{0}}$, *j_appl_* is the applied current density (A m^−2^), and *j*_lim_ is the limiting current density (A m^−2^).

By the end of all experiments, the COD of every organic is reduced by more than 10 times compared to the initial value. The best result was obtained in the case of oxalic acid: complete mineralization.

The dependence of the ICE on time for all organics under study is shown in Figure 4b. In the case of benzoic acid, the ICE is less than in the case of other organics. This may be due to its relatively high stability. The current efficiency in the case of hydroquinone is varied over time. At the beginning of the experiment, it is about two times higher than the ICE in experiments with other substances. The results are repeated in three independent experiments. After 3 h of the experiment, the ICE for all substances sharply decreases and approaches zero values, which is due to a decrease in the limiting current density in the system as the organic concentration in the solution volume decreases.

### 3.3. Current Mode Optimization

**Figure 5:**
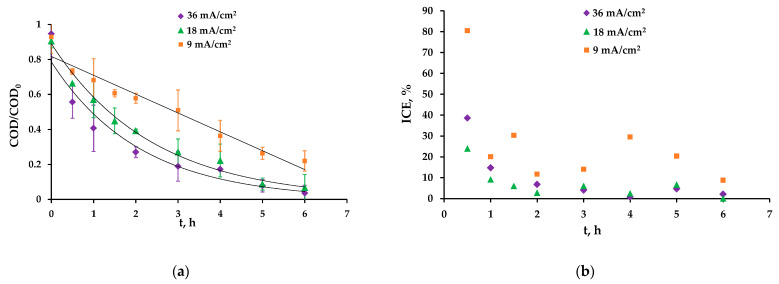
Dependence of the COD/COD_0_ of the hydroquinone solution (**a**) and the ICE (**b**) on the experiment time at different current densities.

To optimize the studied AO process, the experiments were carried out at current densities, respectively, two and four times less than the value used for the oxidation of all organics (Figure 5). In this section, only hydroquinone solutions were used. This substance was selected because hydroquinone and benzoquinone which is formed as a byproduct in the oxidation of hydroquinone, are the most common intermediates in the degradation processes of many aromatic pollutants, such as paracetamol [28].

At lower current densities, the rate of hydroquinone oxidation decreases (Figure 5a). At 18 mA/cm^2^, the exponential dependence of COD on time is preserved, but the COD value at t = 6 h is slightly higher than at 36 mA/cm^2^ and approximately equal to 60 mg/L. At 9 mA/cm^2^, the dependence type changes from exponential to linear. This means that the system is under current-limited control. Such dependence can be described by the linear equation (Equation (4)) obtained in [35]. The COD value reaches 190 ± 11 mg/L by the end of the experiment, which corresponds to the removal of approximately 70% of organics:

(4) $$COD(t)=COD_{0}\left(\frac{1-\alpha Ak_{m}}{V_{R}}t\right)$$

At all current densities, the ICE has the highest value at the beginning of the experiment (Figure 5b). Adsorption could be the reason of such behavior, but we found that there is no sufficient adsorption in our case (see Supplementary Materials, Figure S2). Thus, we attribute this to the rapid oxidation of the hydroquinone to byproducts by hydroxyl radicals and DET after the electric current is applied; the byproducts are more resistant to anodic oxidation, and the ICE decreases with time. At a current density of 9 mA/cm^2^, the ICE approaches 100% at the beginning of the experiment, while at 18 and 36 mA/cm^2^, it is only 47% and 38%, respectively. It seems that the use of pulsed current modes allows the efficiency of the process at high current densities to significantly increase due to the diffusion of the organics from the bulk solution to the anode surface during the pause and its rapid oxidation during the pulse. When the current is zero, organic substances collect near (and may be slightly adsorbed on) the anode surface. Then, when the current is applied, a larger portion of hydroxyl radicals may be involved in the oxidation process than during the constant current mode. 

On average, for the entire time of the experiment, the ICE at current densities of 36, 18 and 9 mA/cm^2^ was 9%, 12% and 25%, respectively.

### 3.4. Pulsed Current Mode

**Figure 6:**
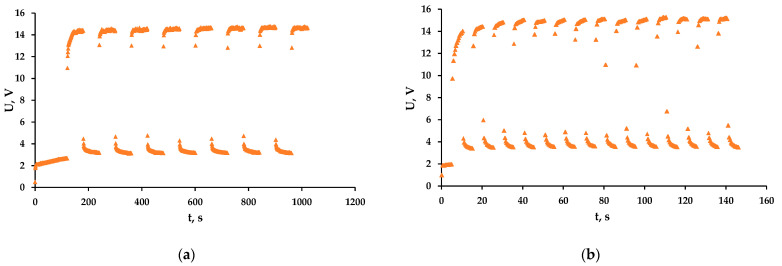
Dependence of the voltage on the experiment time of the hydroquinone solution for the pause/pulse conditions 1 min/1 min (**a**) and 5 s/5 s (**b**) at average time-integrated current density of 18 mA/cm^2^.

The pulsed current modes seem to be promising in different areas of electrochemistry [36,37,38,39]. During the pause, the diffusion of organics to the anode surface occurs, their concentration increases, and when the current is turned on, most of the radicals react with the organic substances. Experiments with a pulsed current mode were carried out with the following parameters: the applied current density during the pulse was 36 mA/cm^2^, the pause/pulse conditions in the first experiment were 1 min/1 min and in the second experiment, 5 s/5 s, respectively. In the 1 min/1 min mode, after the pulse of the current is applied, the voltage in the system increases until it reaches a stationary value (Figure 6a). In the 5 s/5 s mode, the voltage rises for the duration of the application of the pulse (Figure 6b). This is explained by the fact that in 5 sec, the system does not have time to go into a stationary state; that is, diffusion restrictions are reduced compared to the 1 min/1 min mode.

**Figure 7:**
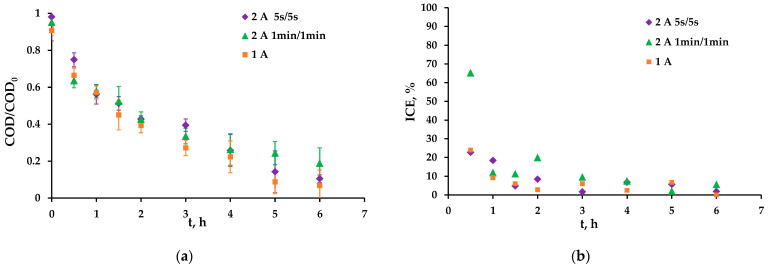
Dependence of the COD/COD_0_ of the hydroquinone solution (**a**) and the ICE (**b**) on the experiment time in pulsed and continuous electrical current modes.

According to the dependencies presented in Figure 7, the use of pulsed current modes did not have the expected effect on the organic oxidation rate. The selected pulsed current modes were compared with the continuous current mode with current density of 18 mA/cm^2^ as the resulting average current density over time is the same both in the pulsed and continuous current mode. The energy consumption for solution pumping was similar in all experiments. In the case of pause/pulse conditions at 1 min/1 min, an insignificant (within the experimental error) deterioration of the AO process is observed: after 6 h, the COD is slightly higher than at a continuous current mode (Figure 7a). At 5 s/5 s, the results are slightly better but also within the experimental error (Figure 7a). The ICE during the pulsed modes also differs little from the continuous current mode (Figure 7b). This probably indicates that the diffusion of organics to the anode surface during the pause period is not a limiting process. The improvement of mass transport conditions due to the use of the pulsed current modes we chose is impossible as the formation of the concentration profile proceeds much faster than the minimum value of the period of the pulsed mode.

### 3.5. Influence of the Hydrodynamic Regime

**Figure 8:**
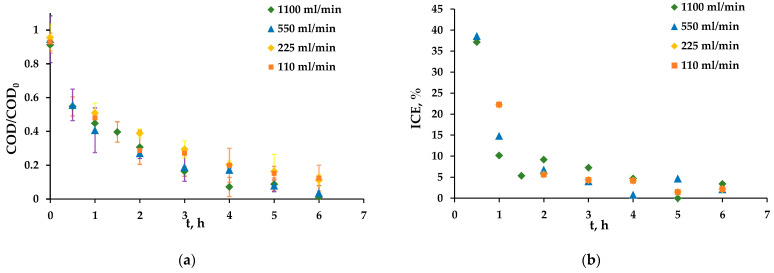
Dependence of the COD/COD_0_ of the hydroquinone solution (**a**) and the ICE (**b**) on the experiment time at different solution flow rates.

The influence of the solution flow rate through the EC-FC was studied. The experiments were carried out at total flow rates of 1100, 550, 225 and 110 mL/min. The effect was observed only at low concentration values after 4 h of the experiment (Figure 8a,b). This is most likely due to the peculiarities of hydrodynamics inside the EC-FC: as the pores inside the separator dividing the cathode and anode are presumably smaller than the pores inside the anode itself, most of the solution could flow directly through the anode. In this case, the transfer of organics to the anode surface close to the cathode, where the main part of HO^•^ radicals is presumably formed [40], is practically independent of the flow rate, and an increase in the solution velocity does not lead to significant changes. Thus, the use of separators with larger pores should increase the organics’ degradation rate.

### 3.6. Anode Degradation

**Figure 9:**
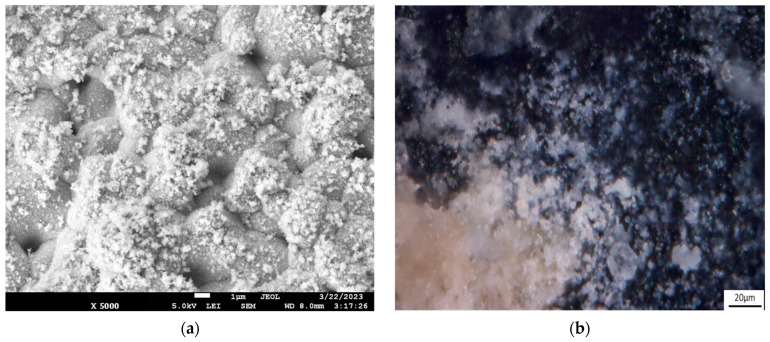
SEM (**a**) and optical images (**b**) of the anode surface after experiments at a current density of 36 mA/cm^2^.

During the experiment at a current density of 36 mA/cm^2^, some solutions of organic compounds showed slight turbidness and became similar to a colloidal solution. In addition, some of the anode granules became covered with a white, water-insoluble precipitate (Figure 9a,b). The proportion of such granules after 108 h of the experiment was less than 0.5%. This indicates a partial degradation of the anode during the experiment. It is possible that the radicals are able to oxidize the anode material, leading to the formation of titanium dioxide (TiO_2_), but we did not find confirmation of this assumption in the literature. The TiO_2_ formation may cause an error in the determination of optical density at a wavelength of 600 nm [41] during the COD determination. Its effect was found and taken into account. A detailed description is given in the Supplementary Materials (see Supplementary Materials, Figure S7). The elemental analysis of the surface composition using SEM-EDS showed that organic substances are present in trace amounts comparable to atmospheric pollution, while Ti and O dominate (see Supplementary Materials, Figure S3). Thus, the assumption that the formed dendrites consist precisely of titanium oxide is the most appropriate. Furthermore, after all series of experiments, some granules (≤0.1%) were covered with a brown precipitate (see Supplementary Materials, Figure S1). At lower current densities, no anode degradation was observed (see Supplementary Materials, Figure S6).

The FT-IR spectra of the surface of the anode granules were obtained before and after experiments (see Supplementary Materials, Figure S5). The obtained spectra are almost identical. This means that for more than 100 h of experiments with various organic substances, no polymer compounds (fouling) were formed on the anode surface, which confirms the data obtained using SEM-EDS.

### 3.7. pH and Conductivity

The electrical conductivity of the freshly prepared solution was 15.5 mS/cm, and after pumping the solution through the EC-FC for 10 min, it decreased to 14.8 mS/cm, i.e., by approximately 4.5% compared to the initial value. This can be explained by the same fact as the change in COD at the beginning of the experiment: the anode and separators, after washing with distillate, retain a small volume of distilled water inside them (approximately 25 mL, 5%). 

**Figure 10:**
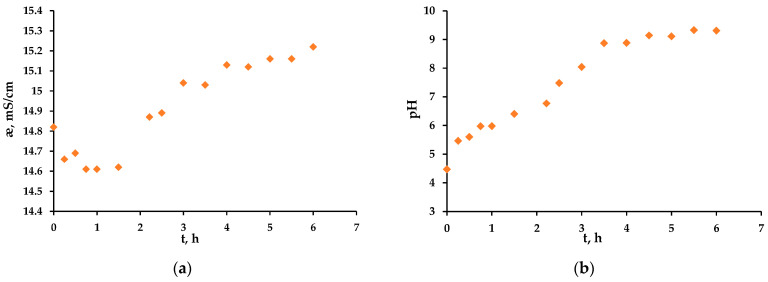
Dependence of the electrical conductivity, æ (**a**) and the pH (**b**) of the hydroquinone solution on the experiment time at the current density of 18 mA/cm^2^.

The electrical conductivity continued to decrease for about 1.5 h after the beginning of the experiment (Figure 10a). After 2 h of the experiment, the electrical conductivity of the solution began to increase, which may be due to the appearance of new charge carriers, such as hydroxyl ions and carboxylic acids, which are formed as byproducts during the oxidation of hydroquinone and are charged in an alkaline medium (Figure 10a).

During the whole experiment, the pH value increased, reaching a plateau by 3.5 h of the experiment and a maximum value of 9.3 by 6 h (Figure 10b). This growth is explained by the higher generation of hydroxyl ions at the cathode compared to the generation of H^+^ ions at the anode, as at the anode, part of the current is spent on the DET reaction. Despite the fact that some of the hydroxyl ions reach the anode surface, donate an electron and become radicals, the rest are retained and increase the resulting pH value of the solution. A high pH value promotes better oxidation of organic matter [42].

## 4. Conclusions

We applied a particle electrode made of Ti_4_O_7_ to the AO of organic pollutants in aqueous solutions. The main advantages of such anodes are high OEP and chemical stability at high pH values. A specially designed electrochemical cell was prepared for the AO process with a particle electrode. It provides organic compounds access to the entire pore surface of the anode.

The high efficiency and stability of the particle Ti_4_O_7_ anode in the process of oxidation of organic acids and hydroquinone are shown. Almost complete mineralization of oxalic acid is achieved after 4 h of the experiment and hydroquinone after 6 h. The use of these anodes may significantly reduce capital costs for electrochemical oxidation systems due to the low cost of the anode material, as well as save on energy costs and electrode regeneration.

## Figures and Tables

**Table 1:** Identified impurities in the Ti_4_O_7_ granules.

No.	Impurities	Mass Content, %
2	SiO_2_	<1.30
3	Al_2_O_3_	<0.08
4	K_2_O	<0.04

## Data Availability

Not applicable.

## Supplementary Materials

The following supporting information can be downloaded at: https://www.mdpi.com/article/10.3390/membranes13050521/s1, Figure S1: SEM and optical images after experiments at a current density of 36mA/cm^2^ of covered anode surface with white water-insoluble precipitate (**a**,**c**) and (**b**,**d**) brown precipitate; Figure S2: Dependence of COD/COD_0_ on the contact time of the anode material with a solution containing 600 mg COD/L and 0.1 M Na_2_SO_4._; Figure S3: SEM-EDS spectrum and SEM images of SEM-EDS analysis areas of anode granules surface after experiments at a current density of 36 mA/cm^2^. The granule without any color changing (**a**,**b**), with white (**c**,**d**) and brown (**e**,**f**) water-insoluble precipitate on the surface; Figure S4: XRD pattern of pristine Ti_4_O_7_ granules for anode material; Figure S5: FT-IF spectra of anode granules before (**a**) and after (**b**) experiments; Figure S6: SEM image of anode surface after experiments at a current density of 9 mA/cm^2^; Figure S7: Optical images of the separator at one- (a,c) and five-fold (b,d) magnification of the image (a and b - the anode side, c and d – cathode side).
