# Supplementary material for: Electrochemical Oxidation of Organic Pollutants in Aqueous Solution Using a Ti4O7 Particle Anode
Source: Membranes (Basel). 2023 May 17;13(5):521. doi: 10.3390/membranes13050521 (PMC10221148; doi:10.3390/membranes13050521)
Supplement: Supplementary file 1 [file membranes-13-00521-s001.zip › membranes-2335256-supplementary.pdf]

membranes

Supplementary Materials

Electrochemical oxidation of organic pollutants in aqueous solution using a Ti4O7 particle anode

Andrey Kislyi, Ilya Moroz, Vera Guliaeva, Yuri Prokhorov, Anastasiia Klevtsova and Semyon Mareev*

Membrane Institute, Kuban State University,149 Stavropolskaya St., 350040 Krasnodar, Russia;
* Correspondence: mareev-semyon@bk.ru; S. Mareev, tel.: +7-961-521-07-35

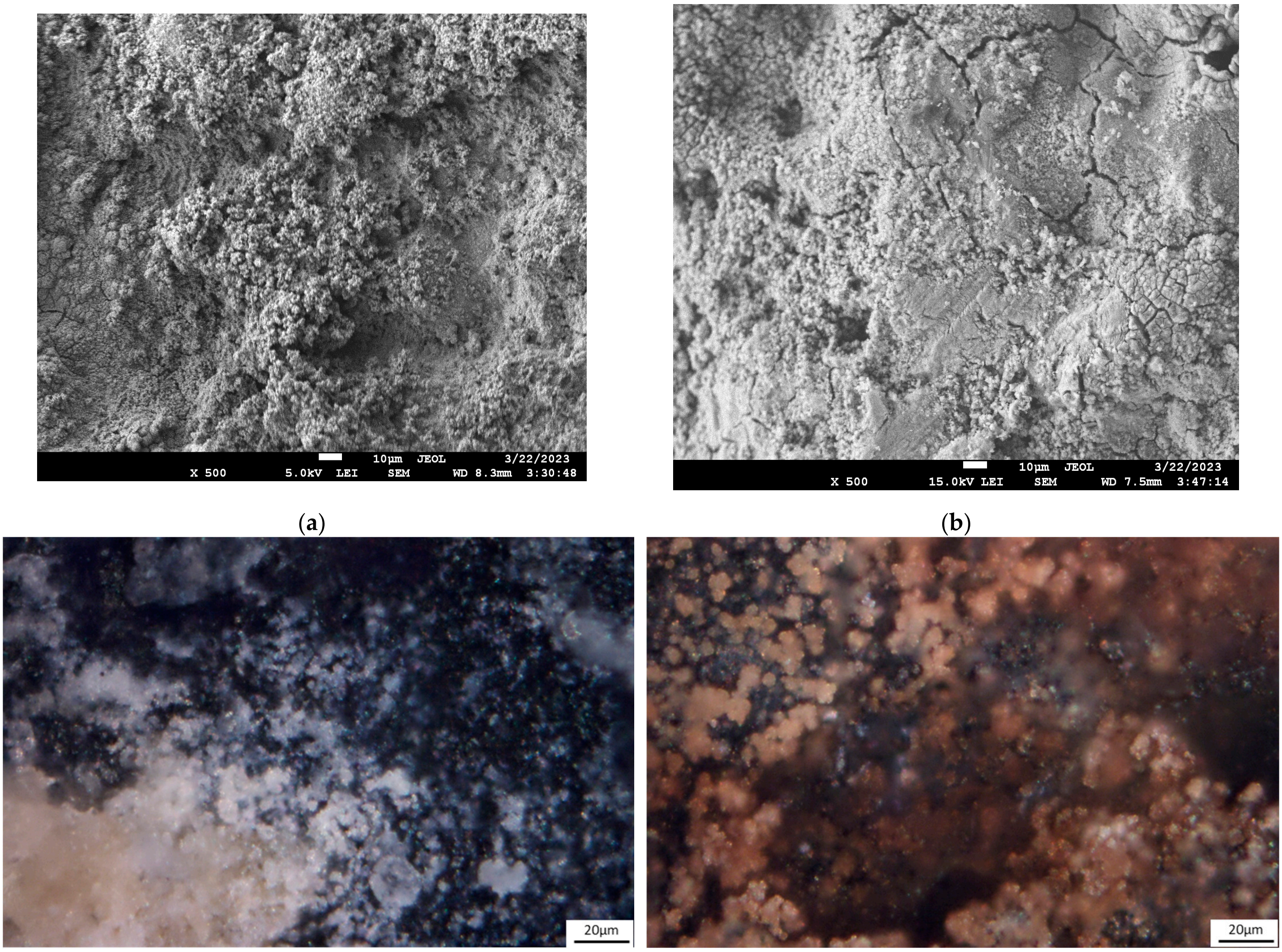

(**a**) (**b**) (**c**) (**d**)

Figure S1. SEM and optical images after experiments at a current density of 36mA/cm² of covered anode surface with white water-insoluble precipitate (**a**,**c**) and (**b**,**d**) brown precipitate.

Adsorption test

Since the anode material has a very developed surface, it is necessary to investigate the possibility of adsorption of organics on it. To test this hypothesis, the following experiment was carried out: a sample of Ti_4O_7 (m = 50 g) was brought into contact with a solution (volume = 0.25 L) containing Na_2SO_4 (c = 0.1 M) and hydroquinone (c = 600 mg COD/L). The solution was continuously mixed with a magnetic stirrer. Samples of the solution in which the COD value was measured were taken at t = 10, 30, 60, 120, and 180 min. According to Figure S2, COD does not change with time. This indicates the absence of significant organic adsorption on the anode surface.

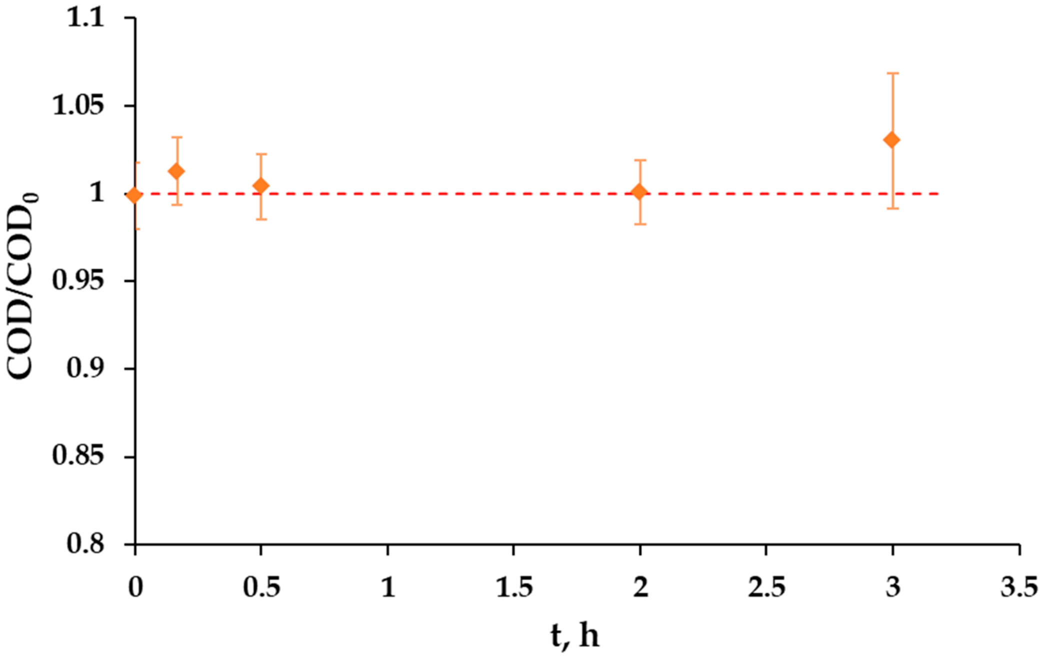

Figure S2. Dependence of COD/COD_0 on the contact time of the anode material with a solution containing 600 mg COD/L and 0.1 M Na_2SO_4.

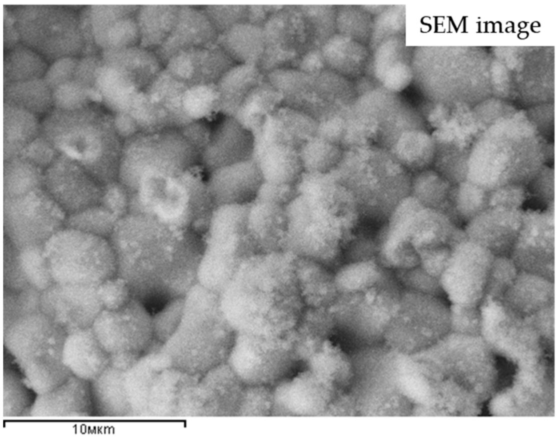

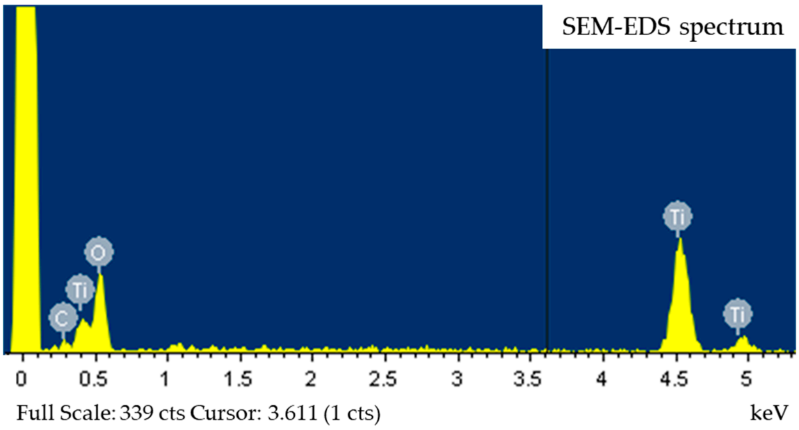

(**a**) (**b**)

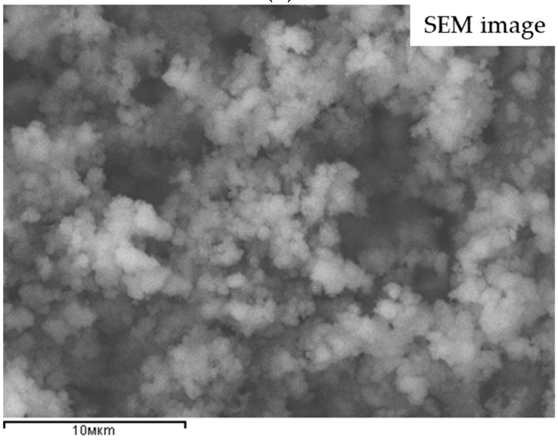

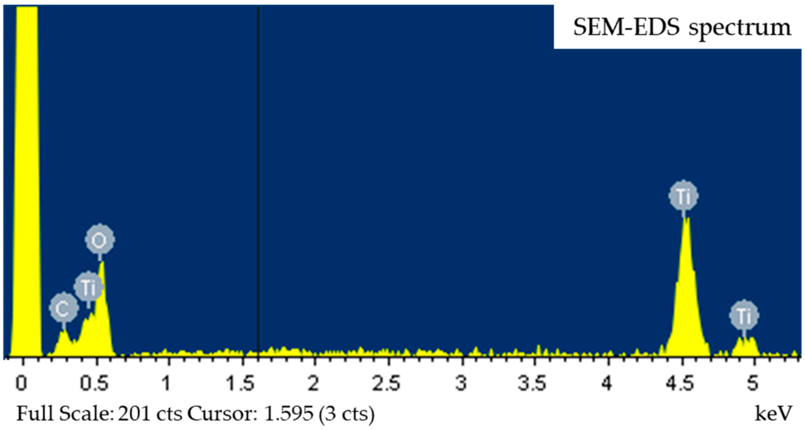

(**c**) (**d**)

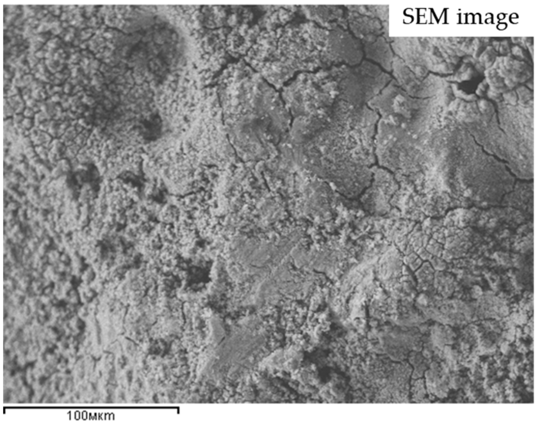

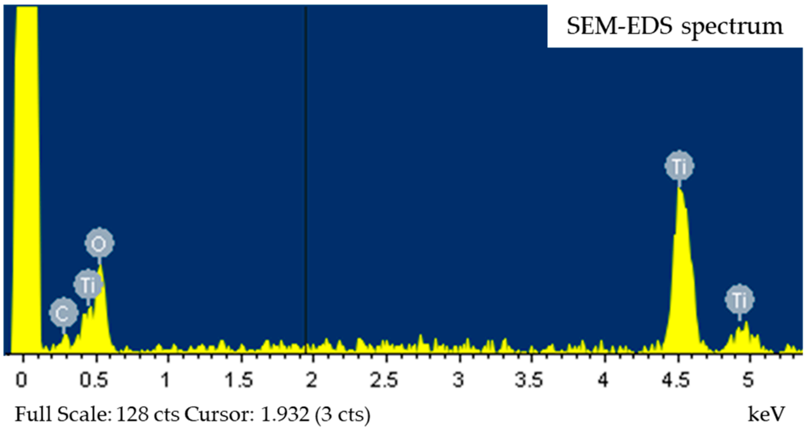

(**e**) (**f**)

Figure S3. SEM-EDS spectrum and SEM images of SEM-EDS analysis areas of anode granules surface after experiments at a current density of 36 mA/cm^2. The granule without any color changing (**a**, **b**), with white (**c**, **d**) and brown (**e**, **f**) water-insoluble precipitate on the surface.

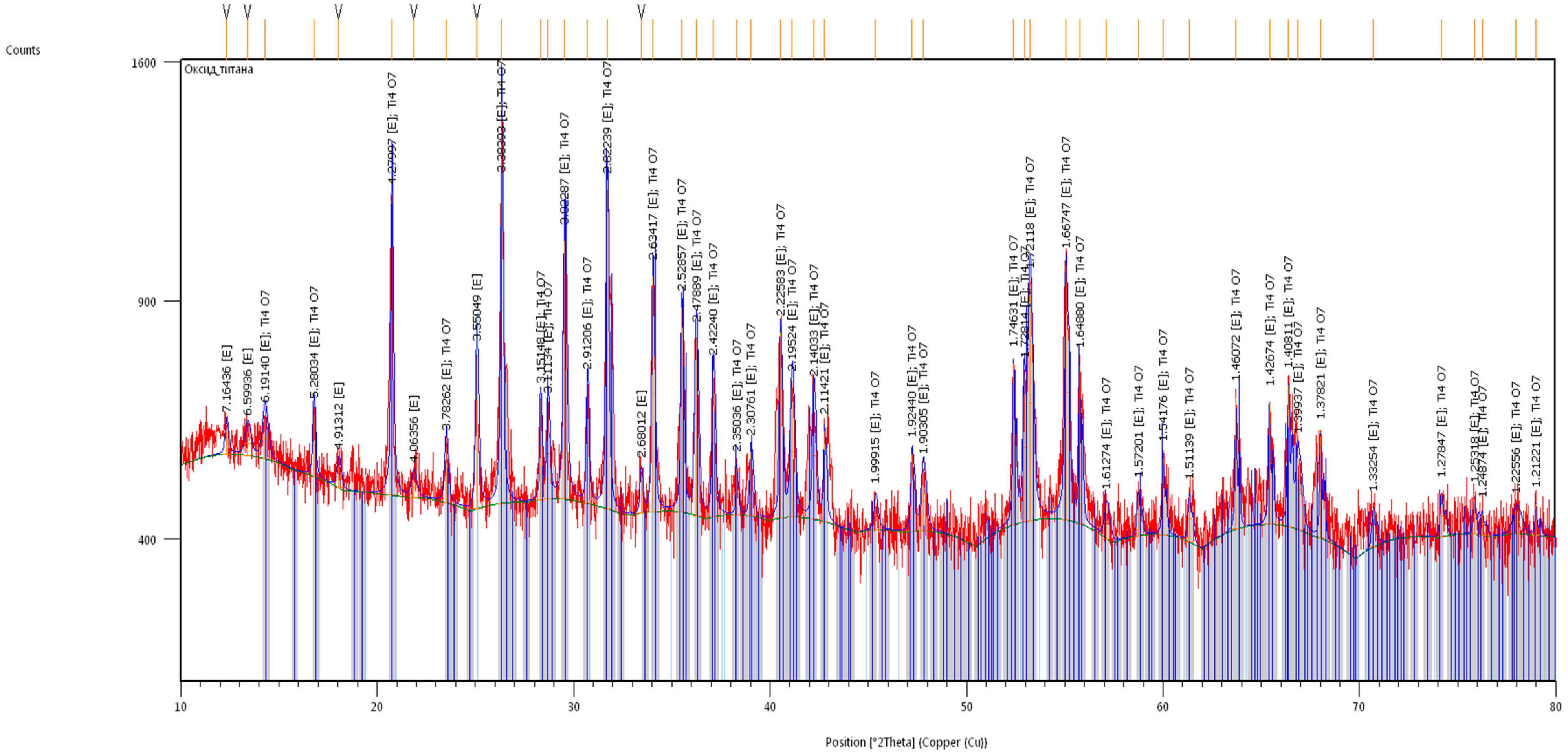

Figure S4. XRD pattern of pristine Ti_4O_7 granules for anode material

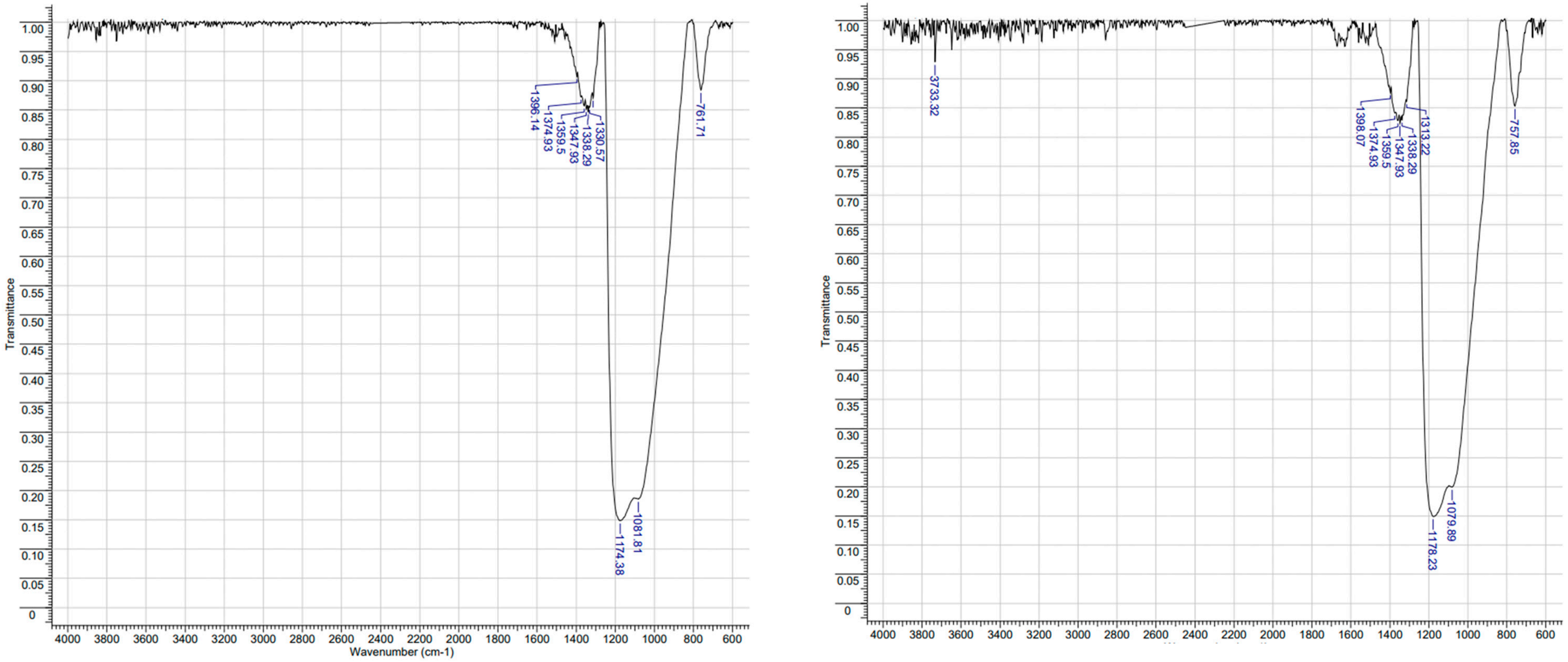

(**a**) (**b**)

Figure S5. FT-IF spectra of anode granules before (**a**) and after (**b**) experiments.

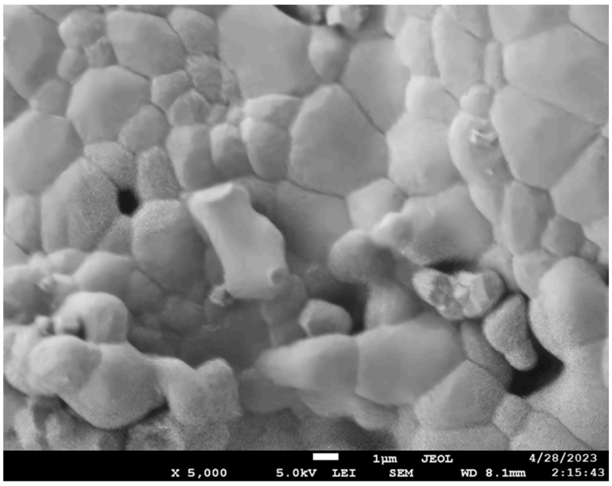

Figure S6. SEM image of anode surface after experiments at a current density of 9 mA/cm^2

Estimation of COD background value. Establishment of the true error of the technique and adjustments

In order to identify the effect of the degradation products of the experimental setup (materials of the case, separator and electrodes) on the quantitative indicators of the COD determination method, a set of experiments was carried out without the addition of any organic substances to the solution, but in the presence of a 0.1M sodium sulfate. During the experiment at a current density of 36 mA/cm^2, the determined value of COD increases with time and reaches a value of 35 mg COD/L at t = 6h.

We attributed this to two possible reasons: 1) destruction of the polypropylene separator and/or case of electrochemical cell, which is in contact with the anode material, followed by fragmentation of the polymer chains and their entry into the solution; 2) destruction of the anode and the formation of TiO_2 nanoparticles during the experiment, which during the implementation of the method for determining COD (with the addition of sulfuric acid and heating) form $TiOSO_4$, which may cause an error in determining the optical density at a wavelength of 600 nm [1].

After more than 100 hours of operation of the experimental setup at a current density of 36 mA/cm^2, no significant degradation of the separator was observed (Figure S7). The formation of a small amount of white and brown deposits on the separator on the anode side was found, which are identical to the deposits on the anode granules. On the cathode side, the deposit on the separator was not observed. A noticeable change in the structure and shape of the separator (polypropylene), as well as the surface of the cell body (teflon) was not detected. It should also be noted that if the detected background COD value were provided by plastic degradation products, more than 300 mg of the separator would have to be dissolved during the entire experiment time (100 hours), which is more than 10% of its total mass. Thus, plastic degradation is not the cause of the observed COD background value. Most likely, the increase in COD is associated precisely with the appearance of titanium dioxide particles in the solution. Analysis of the solution showed its trace amounts. Based on this, we assume that the continuous degradation of the anode leads to the appearance of trace amounts of titanium oxide, which in the process of COD measurement after reaction with sulfuric acid becomes $TiOSO_4$, and we subtract the value determined in these additional experiments from the experimentally determined COD values.

Ion chromatograph with conductometric detector Dionex ICS-3000 was used to verify the obtained data (after correction) in the case of oxalic and maleic acids. We checked the solutions after 5 and 6 hours of the experiment at 36 mA/cm^2. A good agreement between two technics was found.

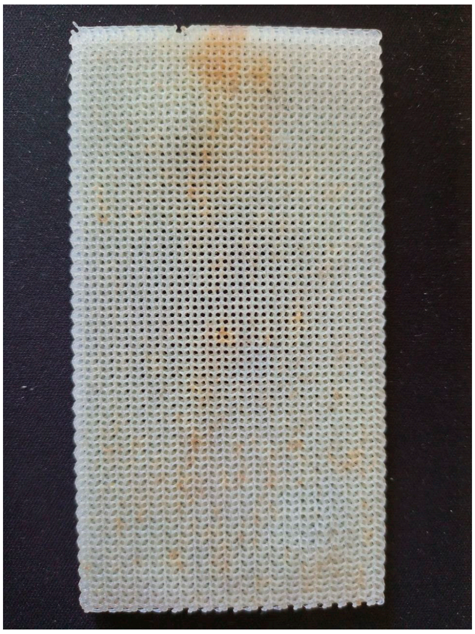

(**a**)

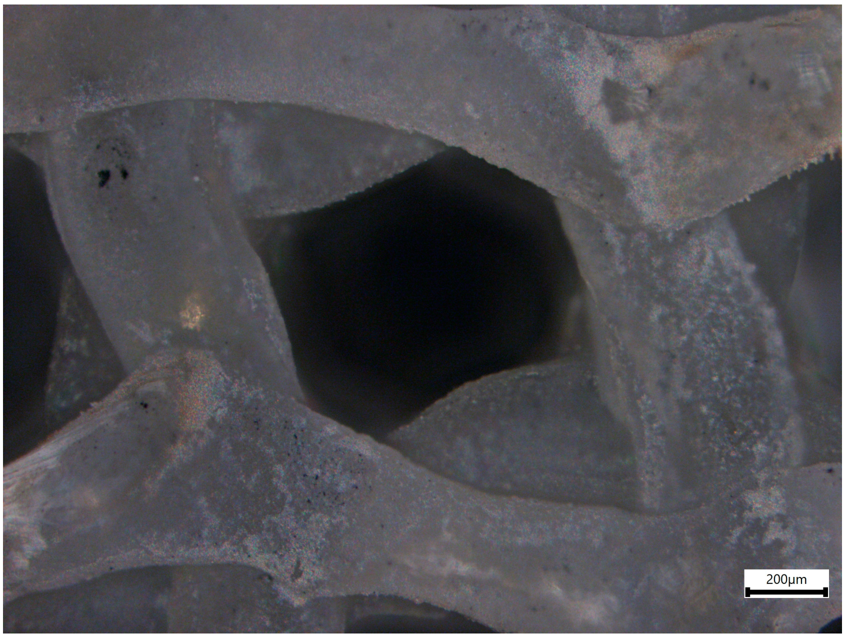

(**b**)

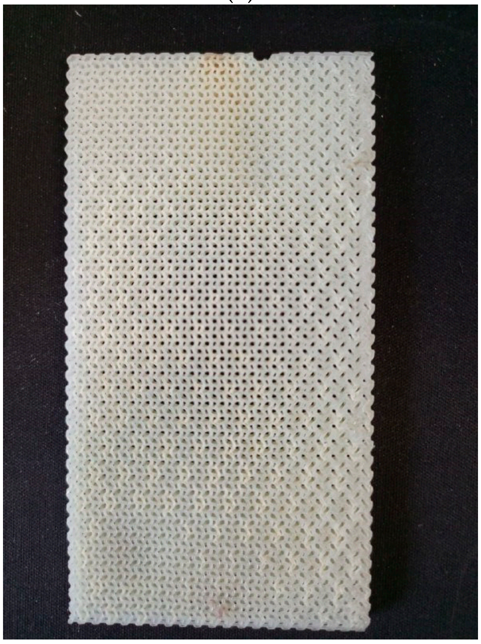

(**c**)

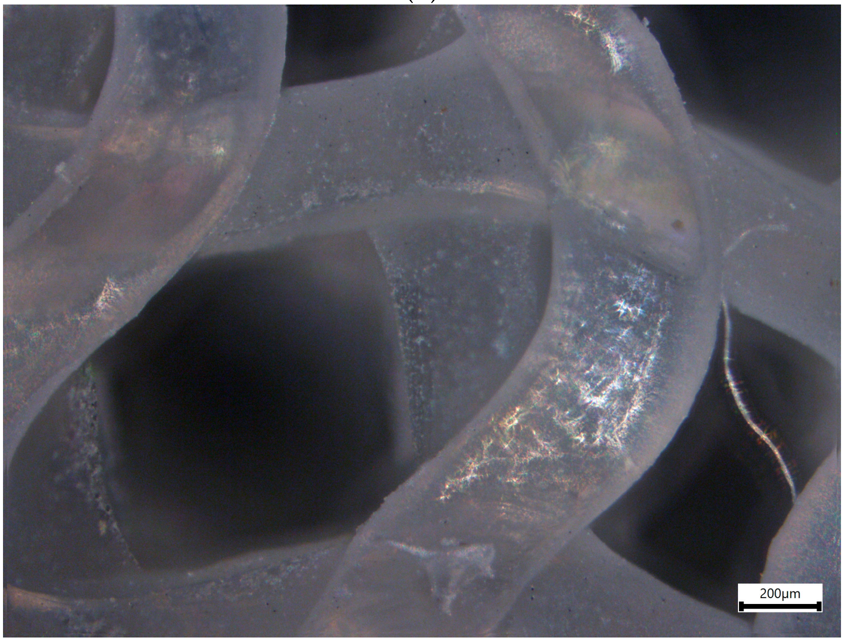

(**d**)

Figure S7. Optical images of the separator at one- (a and c) and five-fold (b, d) magnification of the image (a and b - the anode side, c and d – cathode side).

1. *Enache D.F., Popa G.A., Vasile E., Razvan A., Oprea O., Voicila E., Dumitru F.* // Rev. Chim. 2017. V. 68. No. 11. P. 2635.
